# Maskless X-Ray Writing of Electrical Devices on a Superconducting Oxide with Nanometer Resolution and Online Process Monitoring

**DOI:** 10.1038/s41598-017-09443-3

**Published:** 2017-08-22

**Authors:** Lorenzo Mino, Valentina Bonino, Angelo Agostino, Carmelo Prestipino, Elisa Borfecchia, Carlo Lamberti, Lorenza Operti, Matteo Fretto, Natascia De Leo, Marco Truccato

**Affiliations:** 1 Department of Physics, Interdepartmental Centre NIS, University of Torino, via Giuria 1, 10125 Torino, Italy; 2 Department of Chemistry, Interdepartmental Centre NIS, University of Torino, via Giuria 7, 10125 Torino, Italy; 3 CrisDi Interdepartmental Center for Crystallography, University of Torino, Torino, Italy; 4 Institut Sciences Chimiques de Rennes, UMR-CNRS 6226, Campus de Beaulieu, Université de Rennes 1, 35042 Rennes, Cedex France; 5 IRC “Smart Materials”, Southern Federal University, Rostov-on-Don, Russia; 6 Nanofacility Piemonte INRiM (Istituto Nazionale di Ricerca Metrologica), Strada delle Cacce 91, 10135 Torino, Italy

## Abstract

X-ray nanofabrication has so far been usually limited to mask methods involving photoresist impression and subsequent etching. Herein we show that an innovative maskless X-ray nanopatterning approach allows writing electrical devices with nanometer feature size. In particular we fabricated a Josephson device on a Bi_2_Sr_2_CaCu_2_O_8+δ_ (Bi-2212) superconducting oxide micro-crystal by drawing two single lines of only 50 nm in width using a 17.4 keV synchrotron nano-beam. A precise control of the fabrication process was achieved by monitoring *in situ* the variations of the device electrical resistance during X-ray irradiation, thus finely tuning the irradiation time to drive the material into a non-superconducting state only in the irradiated regions, without significantly perturbing the crystal structure. Time-dependent finite element model simulations show that a possible microscopic origin of this effect can be related to the instantaneous temperature increase induced by the intense synchrotron picosecond X-ray pulses. These results prove that a conceptually new patterning method for oxide electrical devices, based on the local change of electrical properties, is actually possible with potential advantages in terms of heat dissipation, chemical contamination, miniaturization and high aspect ratio of the devices.

## Introduction

The quest for improved device performance has pushed the semiconductor industry to produce smaller integrated circuits, developing lithographic techniques based on radiation with increasingly shorter wavelength^1^. To this aim, the idea of exploiting X-rays, characterized by a very small wavelength (λ ≲ 1 nm), has already been explored in the past, but faced several problems which prevented mass production applications^2^. In this conventional approach the desired pattern should be defined on a photoresist (e.g. polymethyl methacrylate, PMMA), using X-rays to induce some difference in the chemical resistance to the developing solution, and then the pattern defined in the organic material has to be transferred to the electronically active material by the etching process^3^. A first issue is the fabrication of stable masks with the necessary contrast and small sizes. Indeed, masks usually consist of a pattern of a strongly X-ray-absorbing material (typically gold) supported by thin membranes made of silicon nitride, titanium, graphite, or polymers. For transparency reasons, such membranes are only few micrometers thick, but should often extend over big areas making them very delicate^4^. Other relevant problems are represented by the availability of suitable light sources, mask-wafer positioning, and diffraction effects^5^. In this respect, direct-write methods could overcome the limitations inherent to the use of X-ray masks. Moreover, a further simplification of the process can be achieved by avoiding the use of photoresist and the subsequent etching, which can be possible by exploiting the X-rays to directly modify the properties of the material.

In this scenario, the appearance of oxide electronics is opening new possibilities for the application of one-step maskless X-ray patterning methods. The discovery of 2D electron liquids at some oxide/oxide interfaces or the fabrication of oxide-based memory cells show that the oxide technology can play a significant role as an extension and a complement for the silicon one^6, 7^. Since the electrical properties of oxides are very sensitive to the presence of oxygen vacancies in the crystal lattice and since in principle X-rays can directly induce these vacancies, X-ray patterning methods could be suitable to modify the electronic properties of these materials. In this respect, recently the availability of intense nano-focused X-ray beams from synchrotrons and free-electron lasers have dramatically improved the possibilities of using X-rays not only to characterize materials^8–10^, but also to modify their properties in a controlled way^11^, to manipulate defects^12^ and to stimulate the organized growth of nanostructures^13^.

Our group already showed that a high-dose irradiation at 17 keV can affect both structural and electronic properties of Bi_2_Sr_2_CaCu_2_O_8+δ_ (Bi-2212) micro-crystals by modifying their oxygen content^14^. These results are particularly interesting since the existence of a layered structure along the crystal *c*-axis, consisting of alternating superconducting and insulating planes, induces the presence of intrinsic Josephson junctions (IJJs) in this superconducting oxide^15^. The emissions from the one-dimensional array of identical IJJs in crystals of high transition temperature superconductors, such as YBa_2_Cu_3_O_7-δ_^16^, (V_2_Sr_4_O_6_)Fe_2_As_2_^17^ and Tl_2_Ba_2_CuO_6_^18^, have stimulated a new basic and applied research aiming to bridge the “THz gap”. In particular, several studies have been published describing the possibility of producing or sensing coherent THz radiation using Bi-2212 crystals^19, 20^. However, since in Bi-2212 the electrical resistivity along the *c*-axis is about five orders of magnitude higher than in the *ab*-plane^21^, in order to exploit the intrinsic Josephson effect, the current has to be forced to flow along the *c*-axis by patterning the crystal by photolithographic processes or by Focused Ion Beam (FIB) etching^22^, thus introducing vacuum/oxide interfaces at some stage of the process to define the device geometry, which can be detrimental from the point of view of optoelectronics. However, the observation that focused hard X-rays are able to modify the crystal oxygen content^14^ paved the way to the practical realization of a direct-write hard X-ray patterning method where only oxide/oxide interfaces are present, suggesting that a high enough X-ray dose could locally induce in the material an oxygen depletion large enough to drive it in a non-superconducting state. This idea has been recently demonstrated by fabricating a proof-of-concept IJJ device with this novel photoresist-free approach by means of synchrotron radiation, but, in spite of the nanometric sizes of the probe, only micrometric features have been obtained for the devices^23^.

In this contribution we report on the achievement of real nanometric feature size obtained by realizing a IJJ device drawing two single lines of only 50 nm in width. A real-time control of the fabrication process was also achieved by monitoring *in situ* the variations in the electrical resistance of the sample occurring during the patterning procedure.

## Results and Discussion

**Figure 1:**
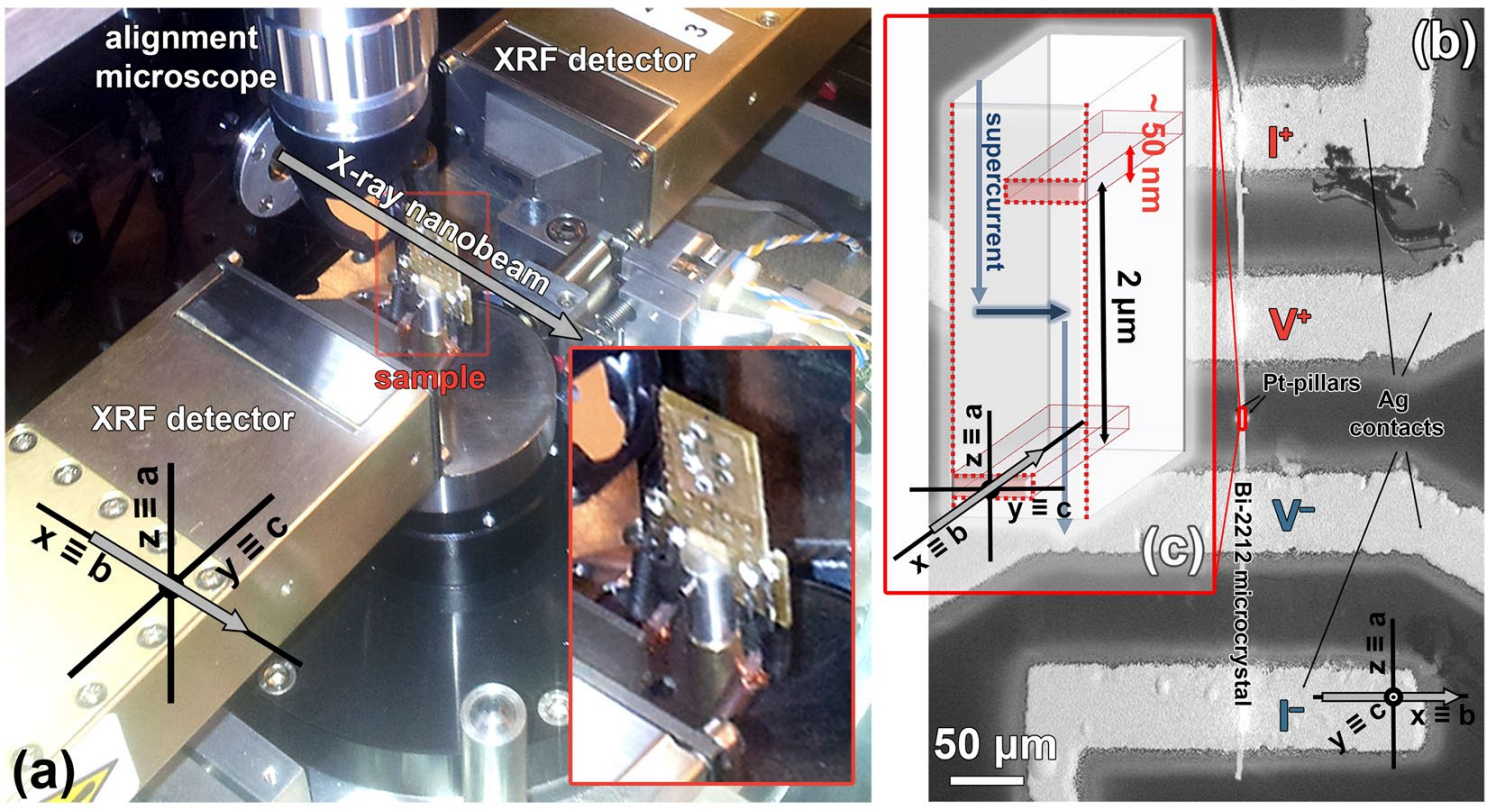
(**a**) Photograph of the experimental setup employed at the ESRF ID16B beamline showing the alignment optical microscope, the XRF detectors and the sample rotation stage around the *z*-axis of the laboratory reference frame. The inset reports a magnification of the sample holder used for the on-line electrical measurements: the chip containing the Bi-2212 micro-crystal is visible in the center of the sample holder. (**b**) Scanning electron microscope (SEM) image of a Bi-2212 micro-crystal mounted on the chip for electrical characterization. The voltage and current Ag electrodes for the on-line four-probes electrical measurements are labeled as V^+^, V^−^, I^+^ and I^−^, respectively. The Pt pillars used for the alignment are visible between the voltage electrodes. (**c**) Schematic representation of the modifications induced by the trenches in the path of the superconducting current, designed to force it along the *c*-axis of the Bi-2212 micro-crystal across a stack of intrinsic Josephson junctions. The reference systems present in all the panels show the relative orientation of the laboratory frame (*x*, y and *z*) and the Bi-2212 crystal axes (*a*, *b* and *c*). The grey arrow represents the X-ray nano-beam.

The first step of the fabrication process was the production of a chip allowing us to measure the electrical properties of the Bi-2212 micro-crystals along specific crystallographic directions. To this aim the micro-crystals were mounted onto sapphire substrates with their *c*-axis normal to the substrate plane and four electrical contacts were realized by Ag physical vapour deposition (see Fig. 1b and also Material and Methods section). To facilitate the location of the region to be patterned by the X-ray nano-beam, two Pt pillars were deposited on the sapphire substrate close to the crystals by Focused Ion Beam (FIB)-assisted chemical vapour deposition. In order to monitor on-line the X-ray patterning process, at the ESRF ID16B beamline^24^ the chip for electrical characterization was mounted on a customized sample holder allowing *in situ* four-probe electrical measurements (see Fig. 1a and also Materials and Methods section).

**Figure 2:**
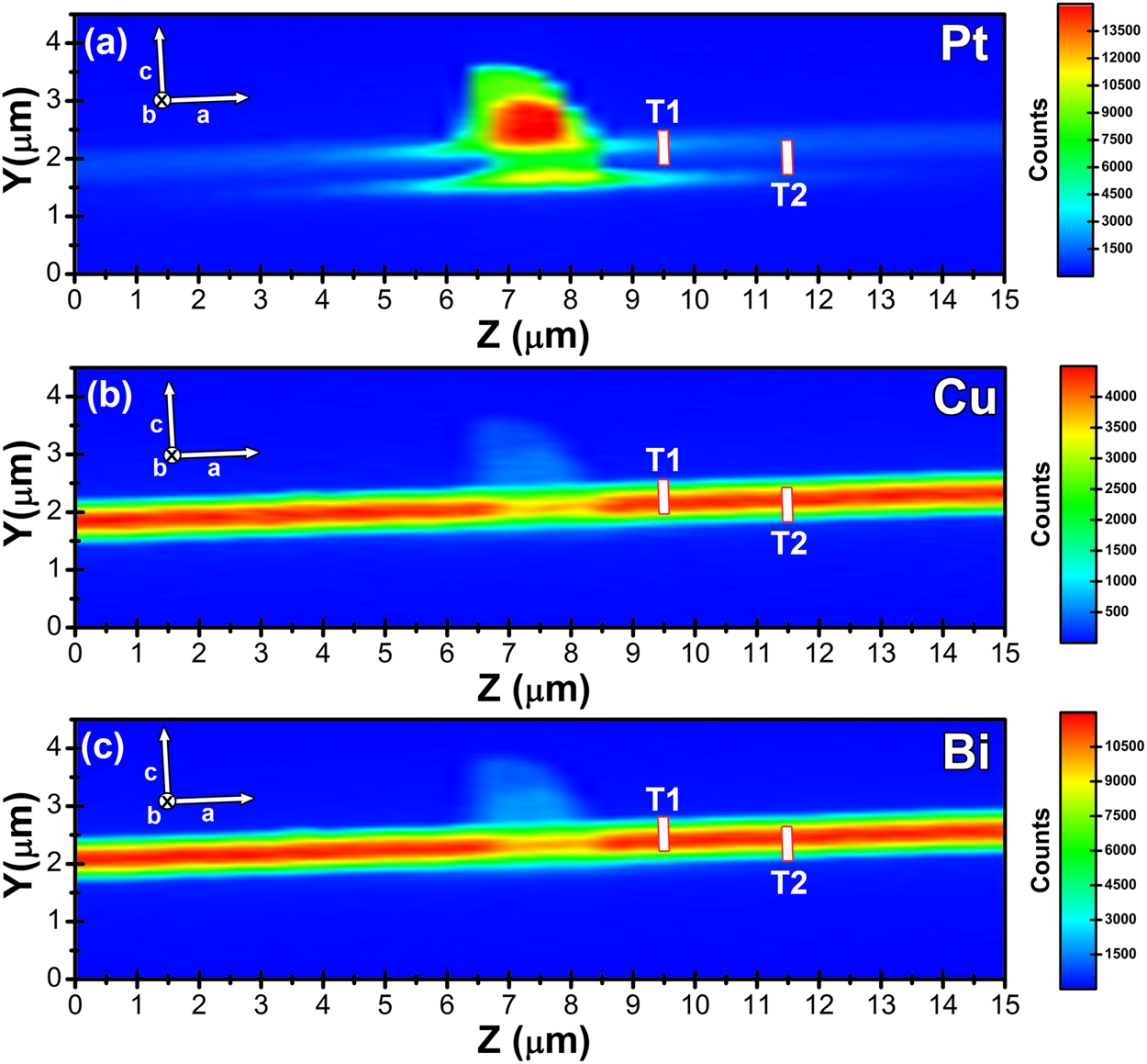
Spatial maps of the integrated XRF counts for Pt Lα (**a**), Cu Kα (**b**), and Bi Lβ (**c**) emission lines. The white rectangles highlight the regions which have been irradiated to realize the first (T1) and second (T2) trenches. The beam is parallel to the [0 1 0] crystallographic direction.

After a preliminary alignment using an optical microscope just to roughly orient the sample so that its [0 1 0] crystallographic direction was parallel to the X-ray beam, the area to be patterned was precisely located exploiting X-ray fluorescence (XRF) maps (Fig. 2) acquired using a 70 × 50 nm^2^ nano-beam at 17.4 keV. These maps will be also employed for the patterning procedure. To avoid sample modifications during the acquisition of the XRF maps the photon flux was opportunely decreased down to 2 × 10^10^ photon/s.

**Figure 3:**
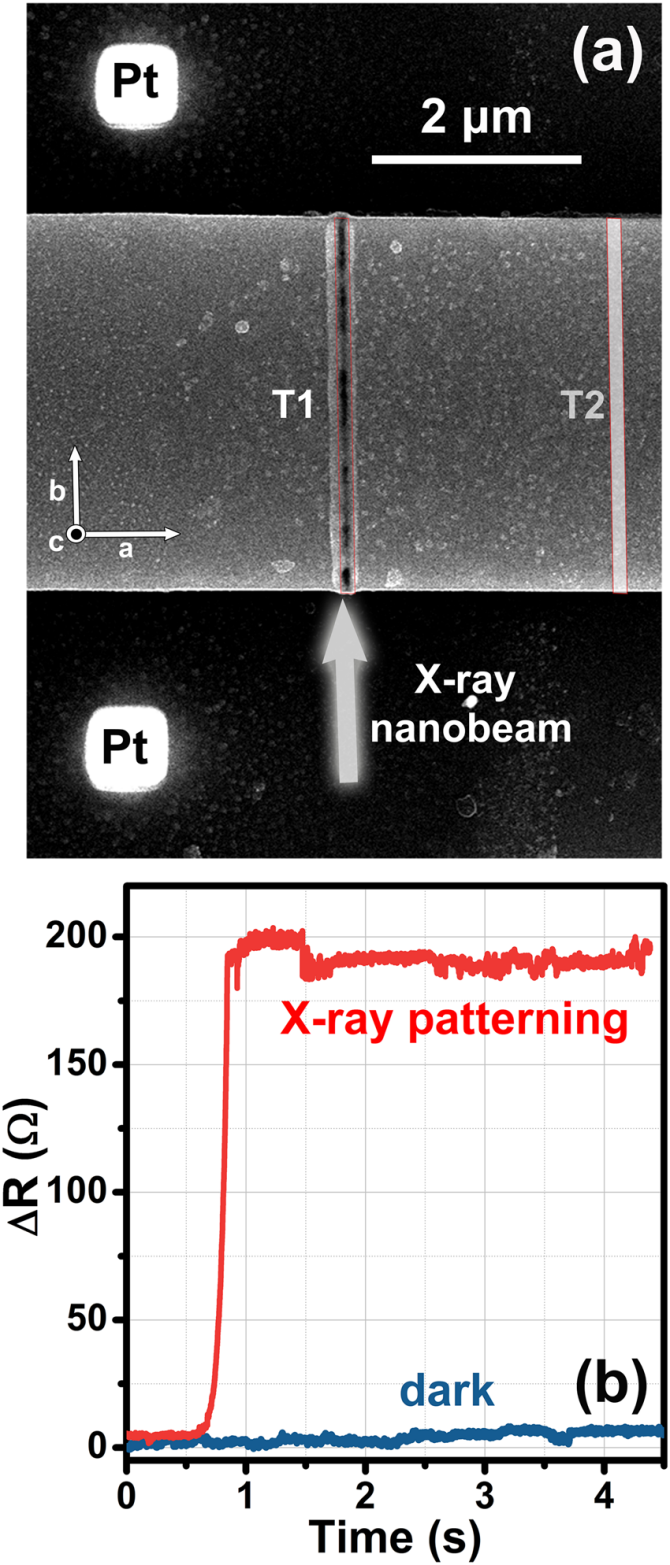
(**a**) SEM image of the Bi-2212 micro-crystal after X-ray irradiation. The white arrow represents the X-ray nano-beam, parallel to the [0 1 0] crystallographic direction, employed to write the two trenches highlighted by red contours. The area irradiated to realize the first trench (T1) is clearly visible, while the area patterned for the second trench (T2), located in the bottom part of the crystal (see also Fig. 2), is highlighted by a white rectangle. (**b**) Variation of the electrical resistance of the sample measured on-line during the X-ray exposure to write the T1 trench (red curve). The blue curve represents the initial sample resistance immediately before the X-ray nanopatterning procedure: its behaviour is indicative of the environmental electrical noise in the on-line measurement setup.

The patterning procedure was performed with a maximum flux of 3 × 10^11^ ph/s by drawing two lines parallel to the sample *c*-axis, thus defining two “trenches”, hereafter referred to as T1 and T2. T1 was drawn in the upper part of the crystal at 2 µm from the centre of the Pt pillars and T2 in the bottom part of the crystal at 2 µm from T1, as visible from Figs 1c, 2 and 3a. This geometry was chosen in order to force the current along the *c*-axis across a stack of IJJs, obtaining a zig-zag path for the current (see Fig. 1c) and thus reproducing the same device geometry that is usually obtained by means of Ga-FIB etching^25^.

In order to finely tune the irradiation time and drive the material into a non-superconducting state just in the irradiated trench regions, the variations in the sample electrical properties during the patterning process were monitored *in situ* by four-probe electrical measurements. As visible in Fig. 3b, during the X-ray exposure to write the T1 trench, the electrical resistance of the sample increased by about 200 Ω. This observation suggests that the X-ray irradiation was effective in modifying the carrier density in the material, which is directly linked to its oxygen content. As already demonstrated in previous studies^26–28^, the non-stoichiometric oxygen content is also related to the critical temperature *T*_*c*_ and the *c*-axis length. In particular, the progressive local oxygen depletion results in an elongation of the *c*-axis and in an increase of both the critical temperature and the normal state resistivity^14^. The possibility to follow on-line the variations of the sample resistance during the exposure to X-rays allows to stop the irradiation as soon as the desired local oxygen depletion is reached, facilitating a fine tuning of the final sample properties.

As shown in Fig. 3a, the trenches can be easily localized using a scanning electron microscope because of a local volume expansion induced by the synchrotron nano-beam. This observation is in agreement with a local oxygen depletion process, which is expected to induce an elongation of the *c*-axis and therefore an expansion along the direction normal to the substrate. Previous nano-XRD measurements highlighted that in this process the local crystallinity of the Bi-2212 is substantially preserved and the only side effect is the possible formation of a layer of polycrystalline α-Bi_2_O_3_ on the crystal surface in the irradiated areas^23^.

**Figure 4:**
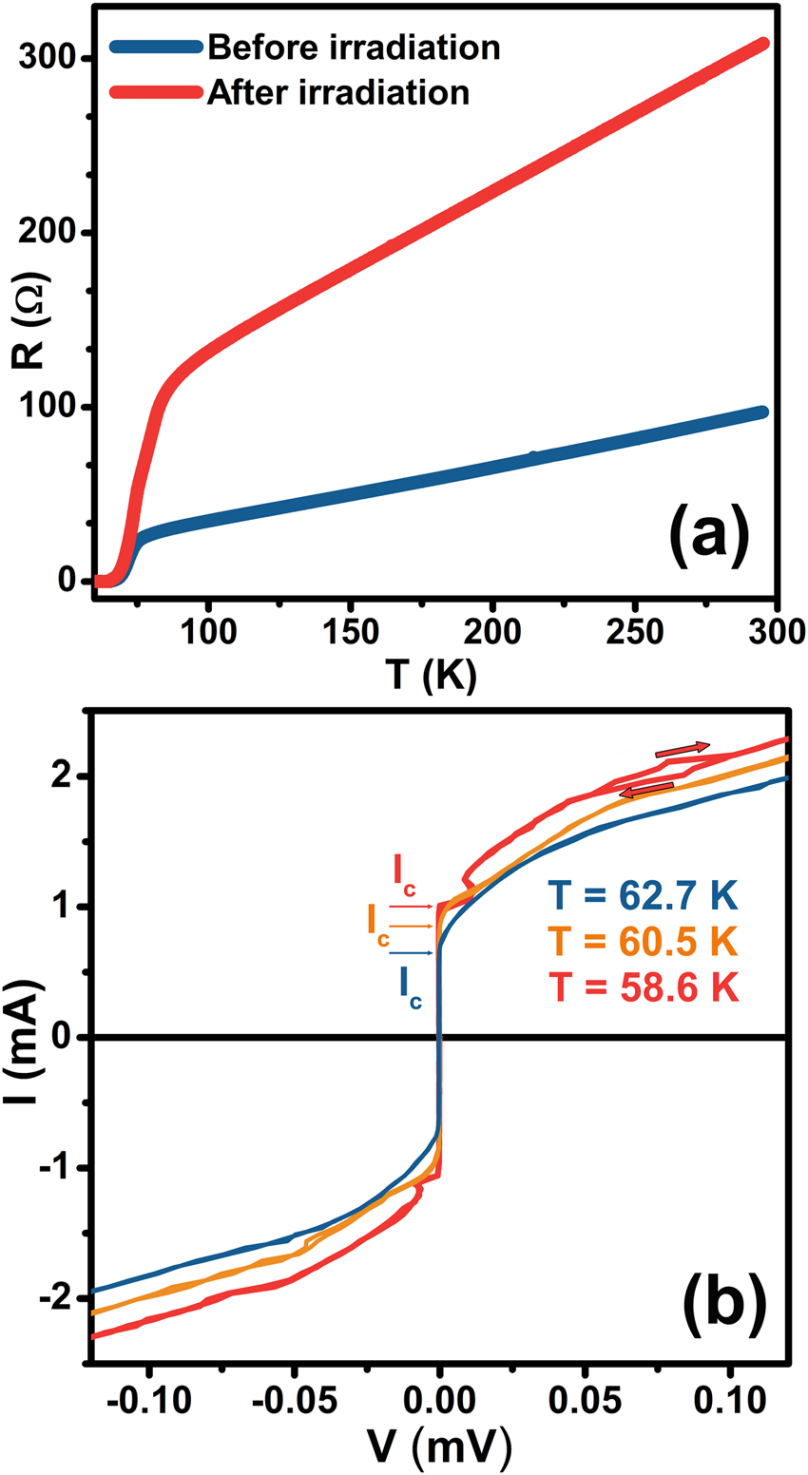
(**a**) Comparison of resistance versus temperature measured in the four-probe configuration between pristine (blue curve) and patterned (red curve) conditions. (**b**) I–V characteristics of a patterned device measured at 58.6 K (red curve), 60.5 K (orange curve) and 62.7 K (blue curve). The red arrows highlight the typical hysteretic pattern in the case of T = 58.6 K. The maximum current amplitudes *I*_*c*_ for the three different temperatures are also indicated.

The resistance versus temperature curves, reported in Fig. 4a, show a remarkable increase in the device resistance at any temperature after X-ray exposure, clearly highlighting a change in its electrical properties. A careful analysis of the temperature region corresponding to the superconducting transition reveals that the temperature *T*_*c*_ of the inflection point changes from 74.2 K in the pristine state to 76.0 K after the patterning process. Since both the *T*_*c*_ and the resistivity values of Bi-2212 are related to its non-stoichiometric oxygen content^21, 29^, these observations confirm that the irradiation in the trench regions has induced local variations of the oxygen content in the crystal.

On the other hand, the device I-V curves reported in Fig. 4b show that zero-voltage branches develop below *T*_*c*_ and that their maximum current amplitude *I*_*c*_ increases with decreasing the temperature. For currents greater than *I*_*c*_ the device enters a resistive state that can also show a hysteretic behaviour, in the sense that the voltage values observed for branches with increasing absolute values of the current are less than the ones corresponding to branches with decreasing absolute values of the current. This pattern clearly identifies the presence of underdamped Josephson junctions that, because of the experimental geometry, correspond to the typical IJJs of Bi-2212.

Therefore, considered as a whole, the results of the electrical characterization (Fig. 4) demonstrate that the X-ray irradiation has driven the doping level of the trench regions out of the superconducting regime, thus forcing the superconducting current to flow along the *c*-axis and giving rise to a stack of IJJs. In agreement with our previous study^14^, this doping change should correspond to a local oxygen depletion that has also increased the normal state resistivity of the irradiated regions, as suggested by the increase of the total electrical resistance of the device (Fig. 4a). Moreover, a possible additional effect could be an increase of the mosaic structure inside the crystal^23^.

A possible microscopic origin of this effect could rely on the high fluence of photoelectrons locally induced by the X-ray nano-beam, which could knock-on the interstitial oxygen atoms of Bi-2212 and change the local conductivity properties of the crystals. This mechanism was already investigated by Piñera *et al*.^30^ in the case of the YBa_2_Cu_3_O_7-δ_ (Y-123) superconductor, showing that 122 keV photons can displace a significant amount of O atoms because of the allegedly low value of the threshold energy *T*_*d*_ for their displacement, assumed to be *T*_*d*_ = 3.45 eV. In the case of Bi-2212, no value for *T*_*d*_ has been explicitly reported so far, to the best of our knowledge. However, both the value of the activation energy for the oxygen diffusion coefficient in the *ab*-plane (0.93 eV)^31^ and the value reported for the binding energy of interstitial oxygen atoms (0.073 eV)^32^ compare favourably to the value used for Y-123, thus suggesting some role of the knock-on mechanism in this effect.

**Figure 5:**
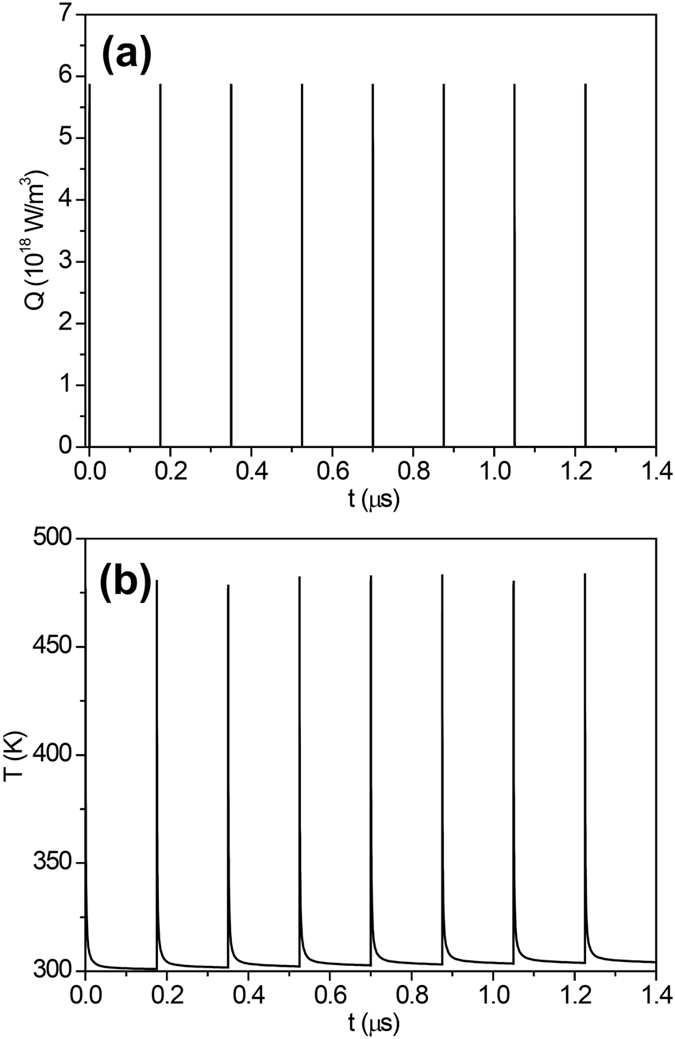
(**a**) Temporal evolution of the heating power density Q at the beam incidence point (the experiment was performed in 16-bunch filling mode). (**b**) Corresponding temporal evolution of the sample temperature calculated at the point of maximum temperature. The initial temperature of the system was set to 300 K.

Another mechanism, which could also be involved, is the local heating induced by the nano-beam. Steady-state finite element model (FEM) simulations have previously ﻿highlighted that in similar experimental conditions the temperature increase is limited to ~23 K^14^. However, transient thermal effects induced by the intrinsically pulsed nature of synchrotron radiation, which could act on very small scales both in space (typical nano-probe size ≲ 100 nm) and in time (typical pulse duration ≲ 50 ps), could significantly modify this picture. To achieve better insights into this issue, we performed improved FEM simulations considering the temporal evolution of the power density absorbed by the sample (see Fig. 5a and Materials and Methods Section). The corresponding temporal evolution of the sample temperature (Fig. 5b) highlights that peak temperatures up to 480 K (i.e. an increase of the temperature of ~180 K) can be reached. This is not in contrast with our previous steady-state results. Indeed, the time integral of the temperature over macroscopic time scales results in an average temperature increase of ~24 K. These findings are particularly interesting since it has already been demonstrated that annealing Bi-2212 micro-crystals at 363 K is sufficient to significantly modify the oxygen content^27^.

## Conclusions

The results discussed in this paper show that fully operational electrical devices can be obtained using direct-write X-ray nanopatterning. This innovative technique is based on the use of hard X-rays to remove just the most loosely bound atoms (interstitial O in our case) from the material, inducing a completely different electrical behaviour without disrupting the crystal structure of the sample. We also showed that the possibility to monitor *in situ* the variations of the device electrical resistance during X-ray illumination ensures a real-time control of the fabrication process, allowing to stop the irradiation as soon as the desired local oxygen depletion is reached, thus facilitating a fine tuning of the final device properties and optimizing the processing time. This approach allowed us to obtain feature sizes in the nanometric domain, realizing an IJJ device by drawing two single lines of only 50 nm in width. In the near future, this limit could be further improved taking advantage of the unique features offered by diffraction limited storage rings^33^ and by X-ray free electron lasers^34^. Finally, we performed FEM simulations of the heating induced by the nano-beam taking into account the time structure of synchrotron radiation, showing that temperatures up to 480 K can be reached. These results provide new insights into the origin of the observed effects, highlighting that the significant instantaneous temperature increase could trigger an oxygen depletion phenomenon.

Possible advantages of this method with respect to FIB etching can be represented by improved mechanical stability, higher thermal conductivity and absence of undesired ion implantation in the fabricated devices. Moreover, this approach allows obtaining the desired device geometry by introducing new material/material interfaces instead of the usual material/vacuum interfaces. This could be exploited to optimize the propagation of electromagnetic waves if the dielectric constant of the irradiated regions can be tuned to suitable values. Another interesting feature, able to offer higher flexibility to the technique, is the high penetration depth of hard X-rays, which can reach tens of micrometers. This peculiarity allows patterning thick layers of material with a high aspect ratio, potentially achieving higher fidelity in the pattern transfer owing to the higher steepness of the device delimiting surfaces with respect to those fabricated by ordinary ion-milling methods.

At the moment, a more extensive use of this technique is hindered by the limited availability of synchrotron radiation sources, although a first automated production line^35^ has already been implemented at the ANKA synchrotron in Karlsruhe. In this respect, the use of phase diffractive optical elements, which can focus X-ray beams into multiple spots or can shape the beam into a desired continuous geometrical pattern, could possibly allow the parallelization of the patterning process^36^. The ongoing developments of ultrabright sources, based on MetalJet X-ray tubes^37^, could also open new possibilities to perform X-ray nanopatterning without the need of synchrotron sources.

In principle, this method could be extended to any oxide whose functional properties are critically influenced by the oxygen arrangement or content, as recently suggested, for instance, for TiO_2_^38^.

## Materials and Methods

### Sample synthesis

The Bi-2212 whiskers were grown starting from high purity commercial powders of Bi_2_O_3_, SrCO_3_, CaCO_3_ and CuO (Aldrich 99.9999%) finely mixed in Bi:Sr:Ca:Cu stoichiometric ratios of 1.5:1:1:2. The mixture was melted at 1050 °C and glassy plates were produced by quenching the melt between copper plates at room temperature. The growth of the crystals took place on the plates during a five day annealing at about 850 °C in a controlled O_2_ flow^39^.

Crystal stability is known to be quite good at room temperature on the time scale of the present experiment^40^. The Bi-2212 sample reported in this paper has a size of 700 × 6.71 × 0.38 *μ*m^3^ (*a*-, *b*-, and *c*-axis directions, respectively).

### Electrical chip preparation

The sample preparation procedure consists in the selection under an optical microscope (100× magnification) of a smooth and linear Bi-2212 crystal which is mounted onto a sapphire substrate with its *c*-axis normal to the substrate plane. Then four electrical contacts are realized by Ag physical vapour deposition (thickness 2.5 µm) and subjected to thermal diffusion at 450 °C for 5 minutes in pure oxygen atmosphere. Pt pillars about 1 µm high were deposited for reference purposes with a FEI Quanta^TM^ 3D FIB (Nanofacility Piemonte, INRIM) at a Ga-ion current of 10 pA.

### Electrical measurements

Each sample was electrically characterized along its *ab* crystallographic plane by the standard four-probe method. Electrical characterization was performed in a continuous flow Janis ST-100 helium cryostat in the temperature range 40 K ≤ T ≤ 290 K. The *R* vs *T* curves were acquired at a constant current value of 1 µA, while for the I-V characteristics the temperature was kept at a constant value and the maximum current was set to 2.5 mA to guarantee device integrity and avoid self-heating phenomena at the current electrodes. For online monitoring, the electrical chips were mounted onto a sample holder compatible with the nano-beam experimental setup and connected to its contact pads by means of Ag wires 50 μm in diameter. The cables of the sample holder were connected to a Keithley 2636 source-meter, choosing a current-source and four-probe voltage-measurement configuration for this instrument. The source-meter was controlled by a computer via a IEEE 488.2 bus, allowing approximately one measurement every 100 ms. DC feeding current for the crystals has been selected between 5 and 100 μA to optimize the signal to noise ratio.

### X-ray nano-beam set-up

The X-ray nanopatterning procedure was performed at the long canted beamline ID16B^24^ at the European Synchrotron Radiation Facility (ESRF) in Grenoble, France. The primary beamline optics (double white mirror and double crystal monochromator) are placed as close as possible to the in-vacuum undulator source to preserve the coherence of the beam, while the nanofocusing optics (Kirkpatrick-Baez mirrors) are located at 165 m, very close to the sample (~0.1 m) to obtain a higher degree of demagnification. The beamline exploits the very low vertical emittance of the ESRF source, and uses a basic principle for the horizontal focusing: a high-β straight section coupled with a horizontal secondary source.

In order to maximize the flux on the sample, we worked in pink beam mode (ΔE/E ≈ 10^−2^), using the double white mirror, but not the double crystal monochromator. The X-ray nanopatterning procedure was performed at 17.4 keV with a maximum flux of 3 × 10^11^ ph/s and beam dimensions at the focal plane of 70 × 50 nm^2^ (V × H) evaluated by the knife-edge scan method^41^, corresponding to an irradiance of 2.4 × 10^10^ mW/cm^2^.

The technical design of the hard X-ray nanoprobe includes an optical microscope to locate the sample and a piezo positioning stage to raster-scan it under the nano-beam. The XRF maps were acquired using an energy dispersive Si drift det﻿ector (see also Fig. 1a). To avoid sample modifications during the acquisition of the XRF maps the photon flux was decreased to 2 × 10^10^ ph/s using a 14 µm Au attenuator and the counting time was set to 0.2 s/point.

### Finite element model simulation of the nano-beam heating effect

To simulate the heating effect of the nano-beam we developed a 3D finite element model (FEM), using the commercial software COMSOL Multiphysics. The heat propagation, including both thermal conduction and convection, has been described by the following equation:

$$\rho {C}_{p}\frac{\partial T}{\partial t}+\rho {C}_{p}u\cdot \nabla T+\nabla \cdot (-k\nabla T)=Q$$

where *T* represents the temperature field, *ρ* is the material density, *C*_*p*_ is the heat capacity at constant pressure, *u* is the air velocity, *k* is the thermal conductivity, and *Q* represents the heat source. Boundary conditions have been assumed by imposing a fixed temperature *T* = 300 K on all the external surfaces of the model. Further details of the model can be found in our previous publications, which report simulations in the stationary state^14, 28^. In this paper, instead, we considered explicitly the pulsed nature of synchrotron radiation and therefore *T* and *Q* are time-dependent.

## References

[1] Seisyan RP (2011). Nanolithography in microelectronics: A review. Tech. Phys..

[2] Marmiroli B, Amenitsch H (2012). X-ray lithography and small-angle X-ray scattering: a combination of techniques merging biology and materials science. Eur. Biophys. J. Biophys. Lett..

[3] Romanato F (2003). Fabrication of 3D metallic photonic crystals by X-ray lithography. Microelectron. Eng..

[4] Maldonado JR, Peckerar M (2016). X-ray lithography: Some history, current status and future prospects. Microelectron. Eng..

[5] Cerrina F (2000). X-ray imaging: applications to patterning and lithography. J. Phys. D-Appl. Phys..

[6] Bibes M, Villegas JE, Barthelemy A (2011). Ultrathin oxide films and interfaces for electronics and spintronics. Adv. Phys..

[7] Jeong DS (2012). Emerging memories: resistive switching mechanisms and current status. Rep. Prog. Phys..

[8] Martinez-Criado, G., Borfecchia, E., Mino, L. & Lamberti, C. In *Characterization of Semiconductor Heterostructures and Nanostructures* (*Second Edition*) (eds Lamberti C. & Agostini G.) 361–412 (Elsevier, 2013).

[9] Ricci, A. *et al*. Multiscale distribution of oxygen puddles in 1/8 doped YBa2Cu3O6.67. *Sci Rep* **3**, doi:10.1038/srep02383 (2013). 10.1038/srep02383 PMC3737503 23924946

[10] Suzuki, Y. & Terada, Y. In *X*-*Ray Absorption and X*-*Ray Emission Spectroscopy: Theory and Applications* Vol. I (eds Jeroen, A. van Bokhoven & Carlo Lamberti) Ch. 10, 251–279 (John Wiley & Sons, 2016).

[11] Poccia N (2011). Evolution and control of oxygen order in a cuprate superconductor. Nat. Mater..

[12] Larciprete R (2002). Direct writing of fluorescent patterns on LiF films by x-ray microprobe. Appl. Phys. Lett..

[13] Malfatti L (2015). Tuning the phase transition of ZnO thin films through lithography: an integrated bottom-up and top-down processing. J. Synchrotron Radiat..

[14] Pagliero A (2014). Doping Change in the Bi-2212 Superconductor Directly Induced by a Hard X-ray Nanobeam. Nano Lett..

[15] Kleiner R, Steinmeyer F, Kunkel G, Muller P (1992). Intrinsic Josephson Effects in Bi_2_Sr_2_CaCu_2_O_8_ Single-Crystals. Phys. Rev. Lett..

[16] Rapp M, Murk A, Semerad R, Prusseit W (1996). c-Axis conductivity and intrinsic Josephson effects in YBa_2_Cu_3_O_7-d_. Phys. Rev. Lett..

[17] Moll PJW, Zhu XY, Cheng P, Wen HH, Batlogg B (2014). Intrinsic Josephson junctions in the iron-based multi-band superconductor (V_2_Sr_4_O_6_)Fe_2_As_2_. Nat. Phys..

[18] Tsvetkov AA (1998). Global and local measures of the intrinsic Josephson coupling in Tl_2_Ba_2_CuO_6_ as a test of the interlayer tunnelling model. Nature.

[19] Ozyuzer L (2007). Emission of coherent THz radiation from superconductors. Science.

[20] Benseman, T. M. *et al*. Direct imaging of hot spots in Bi_2_Sr_2_CaCu_2_O_8+d_ mesa terahertz sources. *J*. *Appl*. *Phys*. **113**, 133902, doi:13390210.1063/1.4795591 (2013).

[21] Watanabe T, Fujii T, Matsuda A (1997). Anisotropic resistivities of precisely oxygen controlled single-crystal Bi_2_Sr_2_CaCu_2_O_8+d_: Systematic study on “spin gap” effect. Phys. Rev. Lett..

[22] Latyshev YI (1999). Interlayer transport of quasiparticles and Cooper pairs in Bi_2_Sr_2_CaCu_2_O_8+d_ superconductors. Phys. Rev. Lett..

[23] Truccato M (2016). Direct-Write X-ray Nanopatterning: A Proof of Concept Josephson Device on Bi_2_Sr_2_CaCu_2_O_8+d_ Superconducting Oxide. Nano Lett..

[24] Martinez-Criado G (2016). ID16B: a hard X-ray nanoprobe beamline at the ESRF for nano-analysis. J. Synchrot. Radiat..

[25] Kim SJ, Yamashita T, Nagao M, Sato M, Maeda H (2002). Development of 3D focused-ion-beam (FIB) etching methods for fabricating micro- and nanodevices. Jpn. J. Appl. Phys. Part 1 - Regul. Pap. Short Notes Rev. Pap..

[26] Inomata K, Kawae T, Nakajima K, Kim SJ, Yamashita T (2003). Junction parameter control of Bi_2_Sr_2_CaCu_2_O_8+d_ stacked junctions by annealing. Appl. Phys. Lett..

[27] Cagliero, S. *et al*. Synchrotron study of oxygen depletion in a Bi-2212 whisker annealed at 363 K. *J*. *Synchrotron Radiat*. **16**, 813–817, doi:10.1107/S0909049509036802 (2009). 10.1107/S0909049509036802 19844018

[28] Mino, L., Borfecchia, E., Agostino, A., Lamberti, C. & Truccato, M. Oxygen doping tuning in superconducting oxides by thermal annealing and hard X-ray irradiation. *J*. *Electron Spectrosc*., in press, doi:10.1016/j.elspec.2016.09.007 (2016).

[29] Triscone G, Genoud JY, Graf T, Junod A, Muller J (1991). Variation of the Superconducting Properties of Bi_2_Sr_2_CaCu_2_O_8+x_ with Oxygen-Content. Physica C.

[30] Pinera I, Cruz CM, Abreu Y, Leyva A (2007). Determination of atom displacement distribution in YBCO superconductors induced by gamma radiation. Phys. Status Solidi A-Appl. Mat..

[31] Runde M (1992). Tracer Diffusion of Oxygen in Bi_2_Sr_2_CaCu_2_O_x_. Phys. Rev. B.

[32] Bandyopadhyay SK (1998). Irradiation-induced oxygen knock-out and its role in bismuth cuprate superconductors. Phys. Rev. B.

[33] Eriksson M, van der Veen JF, Quitmann C (2014). Diffraction-limited storage rings - a window to the science of tomorrow. J. Synchrot. Radiat..

[34] Emma P (2010). First lasing and operation of an angstrom-wavelength free-electron laser. Nat Photonics.

[35] Hahn L, Schwartz G, Saile V, Schulz J (2010). First automated production line for X-ray-LIGA (FELIG) is brought on line..

[36] Di Fabrizio E (2003). Diffractive optical elements for differential interference contrast x-ray microscopy. Opt. Express.

[37] Vagberg, W., Larsson, D. H., Li, M., Arner, A. & Hertz, H. M. X-ray phase-contrast tomography for high-spatial-resolution zebrafish muscle imaging. **5**, doi:10.1038/srep16625 (2015). 10.1038/srep16625 PMC4643221 26564785

[38] Chang SH (2014). X-ray Irradiation Induced Reversible Resistance Change in Pt/TiO_2_/Pt Cells. ACS Nano.

[39] Truccato M (2002). Growth, contacting and ageing of superconducting Bi-2212 whiskers. Supercond. Sci. Technol..

[40] Cagliero S (2009). Crystalline instability of Bi-2212 superconducting whiskers near room temperature. Appl. Phys. A-Mater. Sci. Process..

[41] Takeuchi A, Suzuki Y, Uesugi K (2015). Differential-phase-contrast knife-edge scan method for precise evaluation of X-ray nanobeam. Jpn. J. Appl. Phys..

